# Adult hippocampal neurogenesis: pharmacological mechanisms of antidepressant active ingredients in traditional Chinese medicine

**DOI:** 10.3389/fphar.2023.1307746

**Published:** 2023-12-12

**Authors:** Shimeng Lv, Guangheng Zhang, Yufei Huang, Xia Zhong, Yunhao Yi, Yitong Lu, Jiamin Li, Yuexiang Ma, Jing Teng

**Affiliations:** ^1^ Department of First Clinical Medical College, Shandong University of Traditional Chinese Medicine, Jinan, China; ^2^ Ruijin Hospital Affiliated to Shanghai Jiaotong University School of Medicine, Shanghai, China; ^3^ College of Traditional Chinese Medicine, Shandong University of Traditional Chinese Medicine, Jinan, China

**Keywords:** depression, traditional Chinese medicine, antidepressant, adult hippocampal neurogenesis, pharmacological mechanism

## Abstract

Depression is characterized by prominent indicators and manifestations, such as anhedonia, which refers to the inability to experience pleasure, and persistent feelings of hopelessness. In clinical practice, the primary treatment approach involves the utilization of selective serotonin reuptake inhibitors (SSRIs) and related pharmacological interventions. Nevertheless, it is crucial to recognize that these agents are associated with significant adverse effects. Traditional Chinese medicine (TCM) adopts a multifaceted approach, targeting diverse components, multiple targets, and various channels of action. TCM has potential antidepressant effects. Anomalies in adult hippocampal neurogenesis (AHN) constitute a pivotal factor in the pathology of depression, with the regulation of AHN emerging as a potential key measure to intervene in the pathogenesis and progression of this condition. This comprehensive review presented an overview of the pharmacological mechanisms underlying the antidepressant effects of active ingredients found in TCM. Through examination of recent studies, we explored how these ingredients modulated AHN. Furthermore, we critically assessed the current limitations of research in this domain and proposed novel strategies for preclinical investigation and clinical applications in the treatment of depression in future.

## 1 Introduction

Major depressive disorder (MDD), widely known as depression, represents a psychiatric condition characterized by enduring mood deterioration and diminished capacity for experiencing pleasure. It stands as a significant contributor to global suicide rates. The World Health Organization reports that over 350 million individuals worldwide currently suffer from depression, with an average global incidence rate of approximately 4.4%. By 2030, depression is projected to become the leading disease in terms of global medical burden and serves as the largest non-fatal health loss factor universally (Rehm and Shield, 2019; Bayes et al., 2020). Primary treatment approaches for depression in clinical practice involve the utilization of selective serotonin reuptake inhibitors (SSRIs), which specifically inhibit the reabsorption of 5-hydroxytryptamine (5-HT; serotonin), thereby prolonging and enhancing the effects of serotonin, resulting in an antidepressant response (Perez-Caballero et al., 2014; Bi et al., 2022). However, SSRIs are associated with adverse reactions, including nausea, headaches, sexual dysfunction, and weight gain. Additionally, most treatments encounter issues such as delayed effects and high non-response rates (Wang et al., 2019; Qu et al., 2021; Wei et al., 2022). Therefore, the development of more effective and safer antidepressants has become an urgent concern. Traditional Chinese medicine (TCM) exhibits characteristics such as a multi-component, multi-targeted, and multifaceted nature, making it highly suitable for depression treatment. Certain active ingredients derived from TCM have demonstrated significant antidepressant effects with minimal toxic side effects (Chi et al., 2019), indicating their potential for further research in the field of anti-depression.

Adult hippocampal neurogenesis (AHN) encompasses the entire process of neural stem cell (NSC) proliferation and division within the hippocampus, leading to the formation of neural progenitor cells (NPCs). These NPCs migrate to specific functional regions, undergo plasticity changes and differentiation, and establish synaptic connections with other neurons, ultimately promoting the production of neural function (Kuhn et al., 2018). The relationship between AHN and MDD is of considerable importance, and investigating antidepressant treatments that target the regulation of AHN holds promise for future advancements in antidepressant therapies (Sahay and Hen, 2007). Therefore, it is meaningful to design new treatment strategies for MDD patients and developing depression treatments to regulate AHN.

This review was aimed to provide an academic exposition on the physiological process of AHN and its association with the pathological mechanism of MDD. Additionally, it was also aimed to summarize and analyze the underlying mechanisms through which currently utilized active ingredients in TCM regulate AHN for the treatment of MDD. The objective of this review is to establish a scientific foundation for further basic research and clinical applications in this field.

## 2 Adult hippocampal neurogenesis

The hippocampus is closely intertwined with brain regions implicated in emotion, such as the amygdala and anterior cingulate cortex, and plays a fundamental role in regulating the hypothalamic-pituitary-adrenal (HPA) axis. It is crucial for emotional regulation and for understanding the development of depression (Schumacher et al., 2018; Tartt et al., 2022). Under normal physiological conditions, at least two parts of the adult mammalian brain exhibit sustained neurogenesis. They are the sub vehicular zone (SVZ) located in the later vehicle and the sub granular zone (SGZ) situated in the dentate gyrus (DG) of the hippocampus. Adult hippocampal SGZ NSCs are mainly located between the DG gate and the granulosa cell layer, and are usually in a resting state. When neural stem cells are stimulated, they gradually develop into immature neurons. After a series of processes, they develop into mature neurons, establish synaptic connections with adjacent neurons, and ultimately integrate into the functional neural circuits reflected in the hippocampus (Christian et al., 2014; Yao et al., 2016).

From a microscopic perspective, In the adult hippocampus, NSCs are responsible for generating new neurons. In rodents, NSCs in the hippocampus possess characteristics similar to astrocytes, with radiating protrusions extending to the DG granular cell layer. Therefore, these hippocampal NSCs are commonly referred to as radial glial-like cells (RGL, Type 1 cells). Activation of Type 1 cells results in the production of intermediate progenitors (Type 2 cells). Type 2 cells then differentiate into neuroblast-like cells (Type 3 cells). After several weeks or even months of maturation, Type 3 cells gradually develop into functional granular neurons (Kempermann et al., 2015; Llorens-Martín et al., 2016; Moss et al., 2016; Sánchez-Huerta et al., 2016; Pilz et al., 2018; Li et al., 2021).

## 3 Pathological connection between AHN and MDD

### 3.1 Hippocampal abnormalities

Pathological abnormalities in the hippocampus have been extensively investigated in relation to MDD (Belleau et al., 2019). A study conducted a comparative analysis of Magnetic Resonance Imaging (MRI) results between MDD patients and a healthy control group, unveiling a reduction in the volume of the left hippocampal CA3 and CA4 regions, alongside an elevation in the volume of the right hippocampal amygdala transition area (HATA) (Sun et al., 2023). Another report identified hippocampal atrophy in MDD patients experiencing anhedonia, specifically in the left CA1 and DG subfields, which may be associated with the lack of pleasure endemic to MDD (Wu et al., 2023). Furthermore, MDD patients exhibit diminished Gray Matter Volume (GMV) in the left hippocampus (Brosch et al., 2022). In this investigation, multimodal MRI techniques were employed to scrutinize connectivity patterns in individuals diagnosed with MDD. The findings revealed a significant decrease in the strength of connections within the right hippocampal sub-regional network and the temporal cortex, extending into the insula and basal ganglia. Additionally, the study observed a negative correlation between the degree of depression and functional connectivity (FC) in various brain regions, including the right cornu ammonis 1, right fusion, right HATA, and bilateral basal ganglia (Shengli et al., 2022). Nevertheless, hippocampal volume atrophy is intrinsically linked to a decline in neurogenesis, degeneration of cellular dendrites, and damage to granulosa cell dendrites (Schoenfeld et al., 2017). MDD-related atrophy in hippocampal volume manifests in the brain tissue, resulting in a reduction of hippocampal granule neurons and a decline in the extent of the neurogenic niche. As the brain region of AHN, pathological damage in the hippocampus plays a pivotal role in the progression of MDD. Furthermore, neuropathological damage within the hippocampus serves as a foundation for neurogenic impairment, while angiogenesis and an upsurge in hippocampal volume are vital physiological processes contributing to AHN occurrence (Berger et al., 2020).

### 3.2 Stress and adult hippocampal neurogenesis

Stress is widely acknowledged as a characteristic physiological and psychological response to both favorable and unfavorable circumstances. Prolonged stress constitutes a significant contributing factor in the development of mental disorders, including depression (Mahar et al., 2014). In rodent models, chronic stress is often employed as a model for depression due to its capacity to induce depression-like behaviors such as learned helplessness, anhedonia, and social withdrawal (Schoenfeld et al., 2017). Additionally, stress can inflict damage upon hippocampal neurons (Liu et al., 2021) and cause inflammatory cell infiltration within the hippocampus (Yan et al., 2021), directly or indirectly participating in depression onset. Studies have reported that severe and intense stress can impede AHN within the brain (Cameron and Glover, 2015), while acute or chronic stress during adulthood can hinder the regeneration and survival of new neurons within the DG region of the hippocampus (Garza et al., 2012). Moreover, stress can disrupt AHN by activating the HPA axis pathway and increasing the expression of stress-related hormones (Petrik et al., 2012). Activation of AHN can regulate excessive secretion of the HPA axis and alleviate the stress response (Snyder et al., 2011).

### 3.3 Neuroinflammation and adult hippocampal neurogenesis

Neuroinflammation refers to the inflammatory response occurring within the central nervous system, which can originate from various pathological injuries, including stress, infection, trauma, and ischemia. This process involves the production of pro-inflammatory cytokines, such as interleukin-1β (IL-1β), interleukin-6 (IL-6), and tumor necrosis factor-α (TNF-α), along with reactive oxygen species from innate immune cells within the central nervous system (Leng and Edison, 2021). Neuroinflammation represents a significant pathogenic factor in MDD, as substantial evidence supports the association between depression and the inflammatory process. Inflammation amplifies susceptibility to depression, and the usage of pro-inflammatory drugs heightens the risk of depression among individuals with the disorder (Kohler et al., 2016). Studies have demonstrated that administration of antidepressants reduces peripheral levels of inflammatory cytokines in individuals diagnosed with depression (Liu et al., 2020). The abnormal activation of microglia, resident macrophages within the central nervous system, is responsible for the production of several inflammatory and cytotoxic mediators associated with neuronal dysfunction and brain damage (Woodburn et al., 2021). Microglia express various receptors, including Toll-like receptors (TLRs), with TLR4 being the primary receptor for lipopolysaccharide (LPS). The activation of TLR4 induces downstream transcription factors such as nuclear factor (NF-κB) and the Nod-like receptor pyrin domain 3 (NLRP3), resulting in an increased expression of proinflammatory cytokines and the onset of neuroinflammation (Colonna and Butovsky, 2017). Neuroinflammation can regulate every step of adult neurogenesis, including cell proliferation, differentiation, migration, survival of newborn neurons, maturation, synaptogenesis, and neuritogenesis, when triggered by various immune components such as activated glia, cytokines, chemokines, and reactive oxygen species. Pro-inflammatory cytokines, including IL-6, IL-1β, and TNF-α, can influence the regulation of proliferation, neuronal cell fate, and neuronal differentiation in the context of hippocampal neurogenesis (Green and Nolan, 2014). Additionally, impaired AHN function is closely intertwined with microglial polarization. Stress-induced abnormal activation of microglia can impair the physiological process of neurogenesis, thereby leading to depression-like behavior. Reducing excessive neuroinflammation can ameliorate impaired neurogenesis and serve as a treatment for depression (Amanollahi et al., 2023; Chen et al., 2023).

### 3.4 Role of HPA axis in adult hippocampal neurogenesis

The HPA axis, a pivotal component of the neuroendocrine system orchestrating stress responses, plays a crucial role in the regulation of AHN. Activation of the HPA axis triggers the release of corticotropin-releasing hormone (CRH) from the paraventricular nucleus (PVN) in the hypothalamus, which in turn stimulates the secretion of corticotropin (ACTH) from the anterior pituitary gland. Subsequently, ACTH prompts the adrenal cortex to release cortisol (CORT) into the bloodstream (Frankiensztajn et al., 2020). In patients with depressive symptoms, an overactive HPA axis is associated with elevated levels of CRH, ACTH, and glucocorticoids (GCs), resulting in disrupted negative feedback and consequent pituitary and adrenal gland enlargement, as well as hypercortisolemia (Wang et al., 2021). Research indicates that the excessive activity of the HPA axis inhibits AHN through the activation of glucocorticoid receptors (GRs) and mineralocorticoid receptors by released GCs. However, antidepressant treatments have shown the ability to regulate HPA axis activity and promote AHN (Anacker et al., 2011; Anacker et al., 2013).

### 3.5 Autophagy and adult hippocampal neurogenesis

**FIGURE 1:**
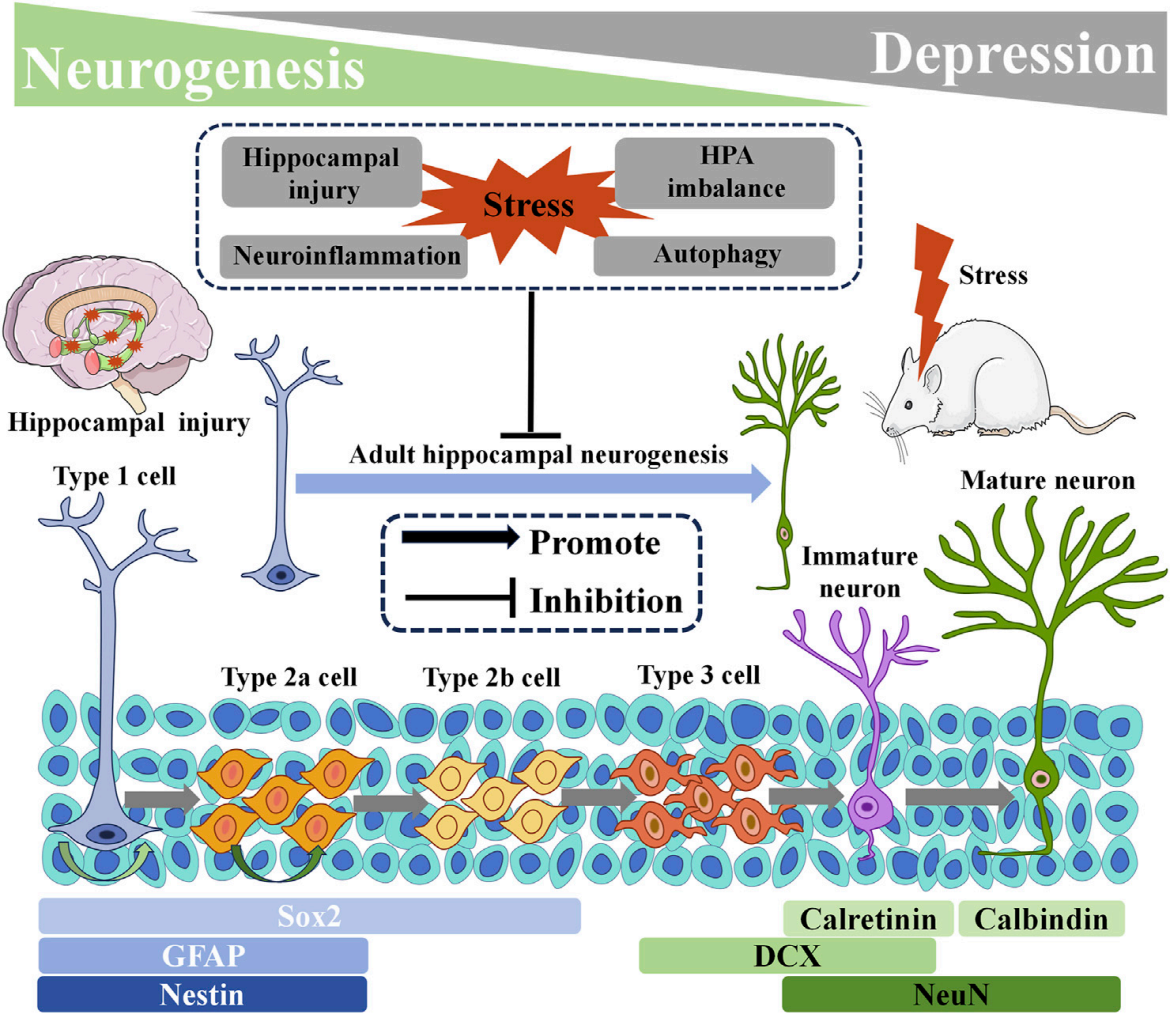
Adult Hippocampal Neurogenesis and Its Relationship with Depression. Below are markers that highlight the specificity of neurogenic processes. HPA: hypothalamic-pituitary-adrenal. DCX: doublecortin. GFAP: glial fibrillary acid protein. NeuN: neuronal nuclei. Sox2: sex determining region Y-box2.

Autophagy, the principal intracellular degradation mechanism responsible for delivering cytoplasmic components to lysosomes for breakdown, serves a broader purpose than mere material removal. It acts as a dynamic circulatory system that generates fresh building blocks and energy, vital for cellular regeneration and maintenance of homeostasis (Mizushima and Komatsu, 2011). Dysregulation of autophagy pathways has been observed in the development of depression, indicating its significant involvement in the pathology of nervous system disorders. Promising results from clinical and preclinical studies targeting autophagy regulation have been reported (Jia and Le, 2015; Gassen and Rein, 2019). Notably, autophagy is closely intertwined with AHN in depression models, wherein chronic stress-induced decline in AHN is mediated by autophagic death of NSCs (Jung et al., 2020). The intervention of CORT triggers the upregulation of autophagy-related gene 5 (ATG5), leading to excessive neuronal autophagy in the DG. It results in heightened degradation of brain-derived neurotrophic factor (BDNF) and a significant reduction in the proliferation of NSCs, NPCs, and neuroblasts. Consequently, the survival and migration of new immature and mature neurons within the DG are impaired. Conversely, downregulation of neuron ATG5 promotes AHN and ameliorates depressive-like behavior in mice (Zhang et al., 2023). Furthermore, the absence of nuclear receptor binding factor 2 (NRBF2), an autophagy-related factor, disrupts autophagy flux in adult NSCs, compromising AHN and inducing a depression-like phenotype. On the contrary, overexpression of NRBF2 in adult NSCs within the DG region mitigates AHN impairment and treats depression (Zhang et al., 2023) (Figure 1).

### 3.6 Effects of antidepressant therapy on adult hippocampal neurogenesis

The hippocampus, as a NSC niche, facilitates neurogenesis throughout adulthood. Dysfunction of the hippocampus due to aging, injury, depression, or neurodegenerative diseases can lead to cognitive decline, significantly affecting the quality of life for individuals. Antidepressant treatments hold promise in directly or indirectly promoting AHN and alleviating depressive symptoms (Kot et al., 2022). SSRIs, commonly prescribed antidepressants, exert their effects by selectively blocking the reuptake of 5-HT, thereby prolonging and enhancing its activity (Perez-Caballero et al., 2014; Bi et al., 2022). Physical activity is another intervention capable of regulating emotional responses and effectively alleviating adverse emotions, including depression (Pearce et al., 2022). Both SSRIs and physical activity have been shown to promote AHN, contributing to their antidepressant mechanisms (Micheli et al., 2018). Recent studies have demonstrated that exercise improves anxiety performance in postmenopausal mice by fostering nerve regeneration in the DG region (Kang et al., 2023). Fluoxetine, a selective SSRI is widely used in clinical practice, ameliorates depression-like behavior by enhancing neurogenesis in a mouse model of Parkinson’s disease (Mendonça et al., 2022a). Additionally, fluoxetine regulates negative behavior during the mouse estrus cycle by increasing AHN (Yohn et al., 2020). Importantly, when normal AHN processes were disrupted using genetic and radiological methods, the therapeutic effect of fluoxetine significantly diminished, highlighting the indispensable role of AHN in antidepressant treatment (Santarelli et al., 2003; Perera et al., 2011). Metformin, a first-line treatment for type 2 diabetes, controls blood sugar levels by suppressing liver gluconeogenesis and affecting glucose metabolism through various mechanisms (LaMoia and Shulman, 2021). It has also been explored for its potential in treating depression. Previous reports suggest that metformin can modulate gut microbiota and autophagy, offering therapeutic benefits for depression. Compared to other oral hypoglycemic drugs, metformin demonstrates a reduced risk of depression and potential efficacy as an antidepressant (Yu et al., 2022; Mendonça et al., 2022b; Yang et al., 2022). Recent studies have revealed that metformin improves depressive-like behavior by promoting AHN (Lv et al., 2023).

In conclusion, a strong correlation exists between AHN and multiple pathogenic pathways associated with MDD. Impaired AHN functionality plays a pivotal role in the development and progression of MDD. Approaches aimed at promoting AHN have exhibited significant therapeutic benefits in preclinical trials for intervening in MDD. Consequently, enhancing AHN has emerged as a prominent area of study for advancing antidepressant medications.

## 4 Mechanism of active ingredients of TCM in promoting AHN in antidepressants

### 4.1 Regulation of the BDNF signaling pathway

BDNF is a growth factor extensively investigated for its involvement in neuronal maturation, synapse development, and synaptic plasticity within the brain (Björkholm and Monteggia, 2016). According to the neurotrophic hypothesis, reduced BDNF expression leads to neuronal atrophy, diminished synaptic plasticity, and contributes to the pathogenesis of depression (van Zutphen et al., 2019). Conversely, optimizing BDNF levels enhances synaptic plasticity and remodeling, mitigates neuronal damage, and ameliorates depressive symptoms (Phillips, 2017). The BDNF/tyrosine kinase receptor B (TrkB) signaling pathway plays a critical role in antidepressant interventions. BDNF facilitates AHN through TrkB regulation, promoting the differentiation and maturation of cortical progenitor cells into neurons during embryonic development (Bartkowska et al., 2007; Donovan et al., 2008). Several studies have reported on the modulation of the BDNF signaling pathway by bioactive components of TCM that foster AHN and alleviate depression.

Oroxylin A, the primary active compound extracted from *Scutellariae radix* (Sajeev et al., 2022), intricately regulates the BDNF/TrKB pathway, fostering AHN and exerting an antidepressant effect (Wu et al., 2022). Camellia assamica var. Kucha (Kucha), a Chinese tea cultivated in Yunnan Province, contains theacrine, a caffeine-like compound and the principal purine alkaloid. It manifests its antidepressant properties by precisely modulating the phosphodiesterase-4 (PDE4)/cyclic adenosine monophosphate (cAMP)/cAMP response element-binding (CREB)/BDNF/TrKB signaling pathway, thereby promoting AHN (Sheng et al., 2020; Ouyang et al., 2021).

Cucurbitacin B, primarily derived from *Cucumis melo L* (Dai et al., 2023), exhibits an antidepressant effect by ameliorating depression-like behavior in mice. Mechanistic investigations have unveiled its involvement in promoting BDNF/TrKB pathway activity and neurogenesis (Ge et al., 2023). Quercetin, abundantly present in various vegetables and fruits, possesses diverse beneficial pharmacological effects (Di Petrillo et al., 2022). In a murine model of depression induced by chronic unpredictable cold stress (CUMS), quercetin administration fosters AHN and treats depression through the Forkhead box transcription factor G1 (FoxG1)/BDNF/TrKB signaling pathway (Ma et al., 2021).

Xanthoceraside, a triterpenoid saponin extracted from *Xanthoceras sorbifolia Bunge* (Zhou et al., 2022), activates the BDNF signaling pathway and AHN, thus alleviating CUMS-induced depression (Guan et al., 2021). Water extracts of Panax ginseng and Polygala tenuifolia also exhibit antidepressant effects by modulating the BDNF/TrKB signaling pathway and promoting AHN (Jiang et al., 2021a). Chronic social distress (CSDS) is often employed in preclinical studies to induce animal models of depression that resemble human depressive mood (Yoshida et al., 2021). Recent reports have demonstrated that CSDS inhibits AHN by impairing the BDNF/TrKB signaling pathway in the hippocampus of mice. However, intervention with Ginsenoside Rb1 can alleviate these pathological phenomena (Jiang et al., 2021b). Another active compound derived from *Panax ginseng C.A. Meyer*, Ginsenoside Rh2, improves depressive behavior in mice by positively modulating the BDNF/TrKB signaling pathway (Shi et al., 2022).

Paeonia lactiflora Pall, a commonly used antidepressant in TCM, contains the water-soluble monoterpene glycoside paeoniflorin, which exhibits various pharmacological activities (Zhou et al., 2020). Recent studies have unveiled that paeoniflorin alleviates CUMS-induced inhibition of AHN by promoting the expression of the BDNF/TrKB signaling pathway (Chen et al., 2019). Echinacoside, a natural phenylethanoid glycoside extracted from *Cistanche Tubulosa* (Li et al., 2022), exerts an antidepressant effect by augmenting the activity of the BDNF/TrKB signaling pathway, regulating M1/M2 polarization of microglia, and inhibiting neuroinflammation (Lu et al., 2023).

Cryptotanshinone, a natural quinone diterpenoid extracted from *Salvia miliorrhiza*, employs the BDNF/TrKB and NFκB signaling pathways to promote AHN and inhibit neuroinflammation[], thus exerting its antidepressant mechanism (Wang et al., 2021). Naringin, a bioflavonoid identified from *Tangerine Peel*, promotes AHN and treats depression by activating the CREB signaling pathway (Gao et al., 2022). Pterostilbene, an active ingredient derived from Dragon’s blood, fosters AHN through the BDNF/extracellular signal-regulated kinase (ERK)/CREB signaling pathway, thereby improving depressive-like behavior in mice subjected to chronic unpredictable stress (CUS) (Yang et al., 2019).

### 4.2 Inhibition of neuroinflammation

Neuroinflammation plays a pivotal role in the pathogenesis of MDD. Excessive neuroinflammatory responses have been shown to hinder AHN, while inhibiting neuroinflammation promotes AHN and ameliorates depressive-like behavior. Thymoquinone, a bioactive compound found in Nigella sativa, effectively suppresses neuroinflammation in the hippocampus and amygdala, promoting AHN and restoring BDNF levels, thus facilitating neurogenesis (Nazir et al., 2022). Hesperidin, a flavanone glycoside abundantly present in citrus fruits such as lemon, sweet orange (Citrus sinensis), and grapefruits (Hajialyani et al., 2019), exerts antidepressant effects through its anti-inflammatory and antioxidant properties, stress reduction, attenuation of cell apoptosis, and enhancement of neurogenesis (Kwatra et al., 2020). Porphyran, an active component isolated from *Porphyra haitanensis*, mitigates exaggerated inflammation induced by LPS in the hippocampus, restores the activity of the BDNF signaling pathway, fosters AHN, and improves depressive behavior in mice (Yi et al., 2021). Patchouli alcohol, the principal active ingredient of Patchouli, inhibits NLRP3 inflammasomes and ameliorates microglia-mediated disturbances in neurogenesis (He et al., 2023). Akebia saponin D, a triterpenoid saponin derived from the rhizome of *Dipsacus asper* (Yang et al., 2021), reprograms neurogenic microglia via the peroxisome proliferator-activated receptor-gamma (PPAR-γ) pathway, rescues hippocampal neurogenesis impaired by Chronic Mild Stress (CMS), and enhances AHN (Zhang et al., 2023). Ginsenoside Rg1 and Ginsenoside Rb1, major components of Panax ginseng C.A. Meyer, exert their antidepressant effects by downregulating neuroinflammation and promoting AHN (Jiang et al., 2020). Similarly, Ginsenoside Rb1 utilizes PPAR-γ mediated activation of microglia to improve AHN in depression treatment (Zhang et al., 2021). Silymarin, a derivative derived from *milk thistle seeds*, has long been used in the treatment of hepatic ailments (Gillessen and Schmidt, 2020). In the treatment of depression, empirical data suggests that Silymarin and Silymarin nanoparticles may exert their therapeutic effects through their antioxidant and anti-inflammatory mechanisms, while also promoting neurogenesis in the prefrontal cortex and hippocampus (Ashraf et al., 2019). Berberine, an isoquinoline alkaloid extracted from the Chinese herb *Coptis chinensis* and various *Berberis* plants, (Song et al., 2020), inhibits NLRP3 inflammasomes to mitigate neuroinflammatory responses, enhances synaptic plasticity and neurogenesis, and improves neuronal degeneration, thereby exhibiting its antidepressant effect (Qin et al., 2023).

### 4.3 Regulation of the HPA axis

The HPA axis, a vital component of the neuroendocrine system, is closely associated with AHN and the pathophysiology of depression. Formononetin, a phytoestrogen obtained from the Chinese medicinal herb *Red Clover* (Yu et al., 2022), promotes AHN by modulating serum CORT levels and hippocampal GR expression in a mouse model of CORT-induced depression (Zhang et al., 2022). Puerarin, a phytoestrogen extracted from *Pueraria* plants (Zhang et al., 2019), holds potential for treating depression-like behavior induced by ovariectomy, with mechanisms involving the inhibition of HPA axis hyperactivity, regulation of BDNF expression, and promotion of AHN (Tantipongpiradet et al., 2019). The Ethanol Extract of Dipterocarpus alatus alleviates HPA axis hyperactivity induced by Unpredictable CMS (UCMS) and regulates BDNF and CREB expression levels (Daodee et al., 2019). The Flower Essential Oil of Tagets Minuta promotes neurogenesis through the modulation of the HPA axis and the BDNF/protein kinase B (Akt)/ERK2 pathway (Birmann et al., 2022). Leonurine, a prominent bioactive constituent derived from *Herba leonuri* (Zhao et al., 2021), promotes axonal growth and neurotrophic activity in cultured PC12 cells through the regulation of the GR/SGK1 signaling pathway (Meng et al., 2019).

### 4.4 Adjustment of the PI3K/Akt signaling pathway

The Phosphatidylinositol 3-kinase (PI3K)/protein kinase B (Akt) signaling pathway represents a crucial regulatory cascade governing cell growth, proliferation, migration, metabolism, and survival (Wang et al., 2022). Targeted modulation of the PI3K/Akt signaling pathway has shown antidepressant effects, as both patients with MDD and animal models exhibit downregulation of PI3K and Akt expression. Moreover, targeted regulation of the PI3K/Akt signaling pathway demonstrates an antidepressant effect (Zhang et al., 2021). Furthermore, this pathway plays a role in promoting AHN by facilitating cellular growth and survival in response to growth factors (Chen et al., 2020). *Xiaoyaosan*, a compound widely employed in TCM, serves as an exemplary representative due to its multiple targets and pathways that contribute to its antidepressant properties (Chen et al., 2022). In a recent study, it was discovered that the ethyl acetate fraction of *Xiaoyaosan* can treat depression by regulating the PI3K/Akt signaling pathway, reducing neuronal apoptosis, and fosters neurogenesis, thereby effectively treating depression (Zeng et al., 2022). The PI3K/Akt signaling pathway also mediates the neuroprotective effect of Akebia saponin D and the antidepressant effects of Baicalin by safeguarding neural stem/precursor cells against inflammatory effects mediated by microglia and stimulating their proliferation and neuronal differentiation, respectively (Liu et al., 2022). *Baicalin*, isolated from *Scutellaria baicalensis*, possesses antidepressant properties due to its association with hippocampal neurogenesis. Previous studies have demonstrated that Baicalin has the ability to modulate the PI3K/Akt/glycogen synthase kinase-3β (GSK3β)/β-catenin pathway, thereby stimulating AHN and eliciting antidepressant effects (Zhao et al., 2020). Moreover, Baicalin has been shown to promote AHN, and alleviate inflammation-induced pain-related depression through Akt-mediated AHN (Fang et al., 2020). Additionally, Baicalin facilitates neuronal differentiation and survival through the Akt/FoxG1 pathway, contributing to its antidepressant effects (Zhang et al., 2019).

### 4.5 Regulation of the microbiota-gut-brain axis

The gut microbiota, an intricate internal metabolic organ comprised of over 10^14^ bacteria and weighing approximately 0.3% of an individual’s body weight, has garnered recognition for its significant role. Emerging research highlights a profound correlation between the gut microbiota and the central nervous system (Xiao et al., 2020). The bidirectional communication between the brain and gut microbiota has captivated scientific interest due to its disruption being identified as a pivotal driver in the development of depression (Du et al., 2020). Notably, the microbiota-gut-brain axis exerts influence on hippocampal neurogenesis by modulating serum metabolite levels (Siopi et al., 2020), while antidepressants have demonstrated efficacy through this axis (Bi et al., 2022).

Eucommia cortex polysaccharides represent the principal active constituents derived from Eucommia cortex (Sun et al., 2022). In a recent preclinical investigation, it was discovered that Eucommia cortex polysaccharides mitigate the release of bacterial-derived LPS, inhibit the TLR4/NFκB/MAPK signaling pathway mediated by microglia, and promote AHN (Wang et al., 2023). Inulin, originally extracted from *Inula helenium*, bestows various beneficial effects upon the body (Illippangama et al., 2022). In a mouse model of chronic unpredictable mild stress (CUMS)-induced depression, disruptions in intestinal microbiota, compromised intestinal barrier integrity, altered levels of short-chain fatty acids (SCFAs), and elevated circulating LPS were observed, resulting in excessive activation of neuroinflammation, impairment of hippocampal neurogenesis, and synaptic plasticity. Inulin intervention ameliorated these pathological phenomena and reversed the depression-like behavior induced by CUMS (Wang et al., 2023). Diosgenin, one of the primary bioactive compounds found in fenugreek seeds (Arya and Kumar, 2021), shows promise in rectifying gut microbiota imbalances, regulating HPA axis secretion levels, upregulating hippocampal BDNF signaling pathway expression, promoting AHN, and treating depression (Cui et al., 2023). Consequently, the microbiota-gut-brain axis assumes a pivotal role for antidepressant interventions, mediating the augmentation of AHN through active ingredients found in TCM.

### 4.6 Regulation of the Wnt/β-catenin signaling pathway

The Wnt/β-catenin pathway plays a critical role in the process of embryonic development and the maintenance of tissue equilibrium in adult organisms. Dysregulation of Wnt/β-catenin signal transduction often accompanies major disorders (Liu et al., 2022). It is widely postulated that Wnt signaling exerts influence on the delicate balance between NSC proliferation and differentiation through transcriptional co-activators, particularly β-catenin, during brain development and adult tissue homeostasis maintenance. Alterations in Wnt signaling have been implicated in developmental abnormalities and neurological diseases. Employing the Wnt/β-catenin pathway as a therapeutic approach for depression and the facilitation of AHN has yielded promising outcomes (Gao et al., 2021).

**FIGURE 2:**
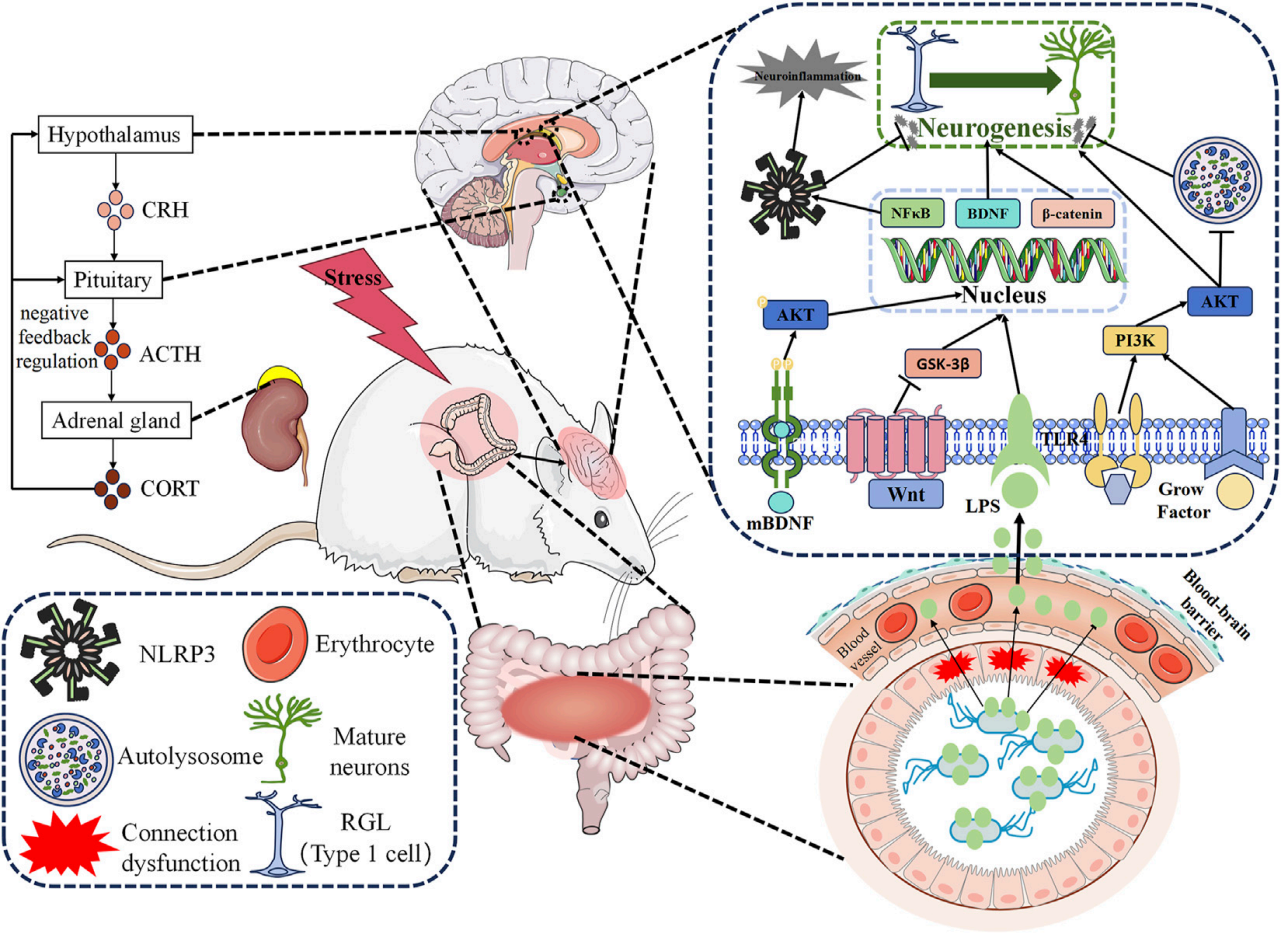
Mechanism of Regulating Adult Hippocampal Neurogenesis by Active Ingredients of Traditional Chinese Medicine in Antidepressant. Active ingredients extracted from Traditional Chinese Medicine promote AHN by regulating various signaling pathways, such as BDNF signaling pathway, PI3K/Akt signaling pathway, and Wnt/β-catenin signaling pathway, as well as by inhibiting neuroinflammation and modulating the HPA axis and microbiota-gut-brain axis. BDNF: Brain-derived neurotrophic factor. PI3K: Phosphatidylinositol 3-kinase. Akt: Protein kinase B. AHN: Adult hippocampal neurogenesis. NF-κB: Transcription factor nuclear factor. NLRP3: Nod-like receptor thermal protein domain 3. CRH: Corticotropin releasing hormone. ACTH: Corticotropin. CORT: Cortisol. LPS: Lipopolysaccharide. RGL: Radial glial-like cells. HPA: hypothalamic-pituitary-adrenal.

Crocin, a hydrophilic carotenoid synthesized in the flowers of the Crocus genus (Boozari and Hosseinzadeh, 2022), has exhibited the capacity to enhance AHN and induce antidepressant effects by modulating the Wnt/β-catenin signaling pathway (Tao et al., 2023). Similarly, Baicalin has demonstrated its ability to counteract depression-like behavior induced by CUMS in mice by finely modulating the Wnt/β-catenin signaling pathway and promoting AHN (Xiao et al., 2021) (Figure 2; Table 1).

**TABLE 1:** Mechanism of action of active ingredients in traditional Chinese medicine.

Active ingredients of TCM	CAS NO.	Molecular formula	Main sources	Modeling method	Behavioral testing evaluation	Mechanism of action/main indicators	References
*Oroxylin A*	480-11-5	C_16_H_12_O_5_	*Scutellariae radix*	CUMS	OFT, FST, TST, SPT	BDNF/TrkB system participates in promoting AHN and antidepressant processes	Sajeev et al. (2022), Wu et al. (2022)
*Theacrine*	2309-49-1	C_9_H_12_N_4_O_3_	*Camellia assamica var. Kucha*	CWIRS + CUMS	FST, TST, SPT, SMAT	Regulating the PDE4/cAMP/CREB/BDNF/TrkB pathway to promote AHN	Sheng et al. (2020), Ouyang et al. (2021)
*Cucurbitacin B*	6199-67-3	C_32_H_46_O_8_	*Cucumis melo L*	CUMS	OFT, FST, TST, SPT	Promote BDNF/TrkB pathway activity and neurogenesis	Dai et al. (2023), Ge et al. (2023)
*Quercetin*	117-39-5	C_15_H_10_O_7_	*Widely distributed in fruits and vegetables*	CUMS	OFT, SPT, TST	Regulating the FoxG1/CREB/BDNF pathway to promote AHN	Ma et al. (2021), Di Petrillo et al. (2022)
*Ginsenoside Rb1*	41753-43-9	C_54_H_92_O_23_	*Panax ginseng C.A. Meyer*	CSDS	SIT, SPT, FST	Enhancing BDNF signaling cascade and promoting AHN	Jiang et al. (2021b)
*Ginsenoside Rh2*	78214-33-2	C_36_H_62_O_8_	*Panax ginseng C.A. Meyer*	CUMS	FST, TST, OFT	Positive regulation of BDNF/TrKB signaling pathway	Shi et al. (2022)
*paeoniflorin*	23180-57-6	C_23_H_28_O_11_	*Paeonia lactiflora Pall*	CUMS	SPT	Promote the expression of BDNF/TrKB signaling pathway to alleviate AHN inhibition caused by CUMS	Chen et al. (2019), Zhou et al. (2020)
*Echinacoside*	82854-37-3	C_35_H_46_O_20_	*Cistanche tubulosa*	CUMS	OFT, FST, TST, SPT	Enhance the activity of BDNF/TrKB signaling pathway and regulate M1/M2 polarization of microglia and inhibit neuroinflammation	Li et al. (2022), Lu et al. (2023)
*Cryptotanshinone*	35825-57-1	C_19_H_20_O_3_	*Salvia miltiorrhiza*	CUS	SPT, FST, FUST, LAT	Through BDNF/TrKB and NFκB signaling pathway to promote AHN and inhibit neuroinflammation	Wang et al. (2021b)
*Naringin*	10236-47-2	C_27_H_32_O_14_	*Tangerine peel*	CORT	TST, OFT, FST	Activating the CREB signaling pathway to promote AHN	Gao et al. (2022)
*Pterostilbene*	537-42-8	C_16_H_16_O_3_	*Dragon’s blood*	CUS	SPT, OFT, FST, NSFT	Promoting AHN through the BDNF/ERK/CREB signaling pathway	Yang et al. (2019)
*Thymoquinone*	490-91-5	C_10_H_12_O_2_	*Nigella sativa*	UCMS	FST, EPM, SIT, NSFT	Inhibiting neuroinflammation in the hippocampus and amygdala and restoring BDNF levels	Nazir et al. (2022)
*Hesperidin*	520-26-3	C_28_H_34_O_15_	*lemon, sweet orange (Citrus sinensis), and grapefruits*	RS + LPS	EPM, OFT, TST, FET, SPT	Anti inflammation, antioxidant stress, alleviating cell apoptosis, and promoting neurogenesis	Hajialyani et al. (2019), Kwatra et al. (2020)
*Porphyran*	11016-36-7	C_26_H_44_O_27_S_2_^−2^	*Porphyra haitanensis*	LPS	FST, TST, OFT	Suppress NFκB/NLRP3 inflammatory signaling pathway, restores BDNF signaling pathway activity, promotes AHN	Yi et al. (2021)
*Patchouli alcohol*	5986-55-0	C_15_H_26_O	*patchouli*	CMS	SPT, TST, FST, OFT, LAT, Coat score	Inhibition of NLRP3 inflammasome and improvement of microglia mediated neurogenic disorders	Lee et al. (2020), He et al. (2023)
*Akebia saponin D*	39524-08-8	C_47_H_76_O_18_	*Dipsacus asper*	CMS	SPT, OFT, FST	Through PPAR-γ Pathway reprogramming of neurogenic microglia to restore hippocampal neurogenesis	Yang et al. (2021), Zhang et al. (2023c)
*Ginsenoside Rg1*	22427-39-0	C_42_H_72_O_14_	*Panax ginseng C.A. Meyer*	CSDS	SIT, SPT, TST, FST	Downregulation and upregulation of neuroinflammation in neurogenesis	Jiang et al. (2020)
*Ginsenoside Rb1*	41753-43-9	C_54_H_92_O_23_	*Panax ginseng C.A. Meyer*	CMS	TST, FST	Through PPAR-γ Mediated activation of microglia and improvement of AHN	Zhang et al. (2021a)
*Berberine*	2086-83-1	C_20_H_18_NO_4_^+^	*Coptis chinensis*	CORT	OFT, TST, FST, SPT	Inhibiting the activation of NLRP3 inflammasomes reduces neuroinflammatory responses and improves neuronal degeneration by promoting synaptic plasticity and neurogenesis	Song et al. (2020), Qin et al. (2023)
*Formononetin*	485-72-3	C_16_H_12_O_4_	*Herb Red Clover*	CORT	SPT, FST, LAT	Reduced serum corticosterone levels, upregulated protein expression levels of GR and BDNF in the hippocampus, and promoted neurogenesis in the hippocampus	Yu et al. (2022b), Zhang et al. (2022)
*Puerarin*	3681-99-0	C_21_H_20_O_9_	*Pueraria plants*	Ovariectomy	TST, FST	Relieve excessive activation of HPA axis and regulate BDNF expression level, promoting AHN	Zhang et al. (2019a), Tantipongpiradet et al. (2019)
*Leonurine*	24697-74-3	C_14_H_21_N_3_O_5_	*Herba leonuri*	CORT	Not Applicable	Regulating the GR/SGK1 signaling pathway	Meng et al. (2019), Zhao et al. (2021)
*Xiaoyaosan ethyl acetate fraction*	Not Applicable	Not Applicable	*Xiaoyaosan*	CUMS	SPT, ST, NFST, TST	Promote hippocampal neurogenesis, reduce neuronal apoptosis, and regulate PI3K/Akt pathway activity	Zeng et al. (2022)
*Akebia saponin D*	39524-08-8	C_47_H_76_O_18_	*Dipsacus asper*	LPS	SPT, FST, EPM, NORT, MWM	Neuroprotective effects are mediated through the PI3K/Akt signaling pathway, protecting neural stem/precursor cells from the inflammatory effects mediated by microglia and stimulating their proliferation and neuronal differentiation	Liu et al. (2022a)
*Baicalin*	21967-41-9	C_21_H_18_O_11_	*Scutellaria baicalensis*	CORT	SPT, OFT, TST, FST, NSFT	Activating AHN and antidepressant effects through the PI3K/AKT/GSK3β/β-catenin pathway	Zhao et al. (2020)
*Baicalin*	21967-41-9	C_21_H_18_O_11_	*Scutellaria baicalensis*	CFA	SPT, TST, Splash test	Alleviation of inflammatory pain related depression through Akt mediated adult hippocampal neurogenesis	Fang et al. (2020)
*Baicalin*	21967-41-9	C_21_H_18_O_11_	*Scutellaria baicalensis*	CUMS	SPT, OFT, TST	Promoting neuronal differentiation and survival through the Akt/FOXG1 pathway, thereby exerting antidepressant effects	Zhang et al. (2019b)
*Inulin*	9005-80-5	Not Applicable	*Inula helenium*	CUMS	TST, FST, OFT, EPM, MBT	Regulate intestinal microbiota disorder and SCFAs levels, protect intestinal barrier, inhibit neuroinflammation, promote AHN, and restore synaptic plasticity	Illippangama et al. (2022), Wang et al. (2023b)
*Diosgenin*	512-04-9	C_27_H_42_O_3_	*Fenugreek seeds*	CRS	SPT, FST	Improve intestinal microbiota imbalance, regulate HPA axis secretion level, upregulate hippocampal BDNF signaling pathway expression, and promote AHN	Arya and Kumar (2021), Cui et al. (2023)
*Crocin*	42553-65-1	C_44_H_64_O_24_	*Crocus genus*	CUMS	SPT, FST, TST	Through Wnt/β-catenin signaling pathway promotes AHN and exerts antidepressant effects	Boozari and Hosseinzadeh (2022), Tao et al. (2023)
*Baicalin*	21967-41-9	C_21_H18O_11_	*Scutellaria baicalensis*	CUMS	TST, EPM, SPT	Adjusting Wnt/β-catenin signaling pathway, activating AHN	Xiao et al. (2021)

Note:CUMS, chronic unpredictable mild stress; CWIRS, chronic water immersion restraint stress; CSDS, Chronic social defeat stress; CUS, Chronic unpredictable stress; RS, Restraint stress; LPS, Lipopolysaccharide; CFA, Freund’s adjuvant; CRS, Chronic restraint stress; SIT, Social Interaction Test; LAT, Locomotor activity test; NSFT, Novelty-suppressed feeding test; ST, Splash test; NORT, Novel object recognition test; MWM, Morris water maze; MBT, Marble burying test; OFT, Open field test; TST, Tail suspension test; FST, Forced swimming test; SPT, Sucrose preference test; SMAT, Spontaneous Motor Activity Test; BDNF, Brain-derived neurotrophic factor; TrKB, tyrosine kinase B; AHN, Adult hippocampal neurogenesis; PDE4, Phosphodiesterase-4; cAMP, Cyclic adenosine mono—Phosphate; CREB, cAMP response-element binding; FoxG1, Forkhead box transcription factor G1; EPM, Elevated Plus Maze; NF-κB, Nuclear transcription factor-κB; NLRP3, Nucleotide-binding oligomerization domain-like receptor protein 3; PPAR-γ, Peroxisome proliferator-activated receptor-gamma; GR, Glucocorticoid receptor; SGK1, Serum-inducible and glucocorticoid-inducible kinase 1; PI3K, Phosphatidylinositol 3-kinase; Akt, Protein kinase B; GSK3β, Glycogen synthase kinase-3b; HPA, Hypothalamic-pituitary-adrenal; SCFAs, Short-chain fatty acids; TCM, traditional chinese medicine.

## 5 Discussion

MDD is a debilitating, chronic, and recurrent mental illness characterized by profound emotional distress, feelings of inadequacy, somatic discomfort, disturbances in sleep or appetite, and an elevated susceptibility to suicidal attempts and actions (Xia et al., 2023). Despite its substantial impact on individuals and society, the pathophysiology of MDD remains enigmatic, and effective interventions are limited, posing an enduring challenge in contemporary medicine (Chen et al., 2022). TCM encompasses a myriad of components, targets, and pathways that harbor potential therapeutic benefits. Moreover, bioactive constituents derived from TCM possess the capacity to engender AHN through diverse signaling pathway, including BDNF, PI3K/Akt, and Wnt/β-catenin, as well as via the modulation of neuroinflammation and the intricate interplay within the HPA and microbiota-gut-brain axes. These bioactive entities hold immense promise for the treatment of MDD.

Nevertheless, current research predominantly relies on animal or cellular models, lacking sufficient exploration into the clinical efficacy of TCM active ingredients in alleviating depression among MDD patients. Additionally, numerous bioactive compounds sourced from TCM encounter challenges such as instability, poor solubility, and limited ability to traverse the blood-brain barrier. The precise targeting of organs implicated in MDD by these bioactive agents also remains uncertain. Furthermore, several Chinese herbal medicines lack well-defined quality control standards, impeding the assurance of chemical component stability and consistency, thereby constraining their clinical utility and hindering the investigation of their pharmacological mechanisms. Most notably, there exists a paucity of research pertaining to the specificity of TCM active ingredients in relation to AHN, warranting further scrutiny to ascertain the ability of these bioactive moieties to efficaciously target AHN.

Therefore, future research endeavors should concentrate on expanding clinical observations regarding the efficacy and adverse reactions of TCM active ingredients in treating MDD patients, while concurrently ameliorating the quality control standards of TCM. Moreover, considerable efforts ought to be devoted to enhancing the exploration of targeted delivery systems for TCM that augment drug concentration and duration of action within the central nervous system, thereby heightening the therapeutic effects of bioactive constituents in target organs. Additionally, the integration of multiple omics techniques can enrich our understanding of the intricate pathways involved in the promotion of AHN by TCM active ingredients, thus fortifying the connection between AHN and MDD. Finally, the incorporation of antagonists or reverse validation methods, such as gene knockout strategies, can facilitate the elucidation of the mechanisms through which Chinese herbal active ingredients regulate AHN. Extensive work would be required in clinical and preclinical studies to unravel the underlying mechanisms by which antidepressant treatments regulate AHN. It is of great significance for the development of TCM as a therapeutic modality for MDD.
